# Influence of Elliptical Fiber Cross-Section Geometry on the Transverse Tensile Response of UD-CFRP Plies Based on Parametric Micromechanical RVE Analysis

**DOI:** 10.3390/ma19020359

**Published:** 2026-01-16

**Authors:** Zhensheng Wu, Jing Qian, Xiang Peng

**Affiliations:** College of Mechanical Engineering, Zhejiang University of Technology, Hangzhou 310023, China; 221125020347@zjut.edu.cn (J.Q.); pengxiang@zjut.edu.cn (X.P.)

**Keywords:** unidirectional CFRP, elliptical fiber cross-sections, transverse tensile response, micromechanics, virtual testing, RVE

## Abstract

Predicting the transverse tensile properties of unidirectional CFRP plies is often based on micromechanical representative volume elements (RVEs) with circular fiber cross-sections, whereas microscopic observations show pronounced ellipticity and size variability in actual fibers. A two-dimensional plane-strain micromechanical framework with elliptical fiber cross-sections is developed as a virtual testing tool to quantify how fiber volume fraction, cross-sectional aspect ratio and statistical fluctuations in the semi-minor axis influence the transverse tensile response. Random RVEs are generated by a hard-core random sequential adsorption procedure under periodic boundary conditions and a minimum edge-to-edge gap constraint, and the fiber arrangements are validated against complete spatial randomness using nearest-neighbor statistics, Ripley’s K function and the radial distribution function. The matrix is described by a damage–plasticity model and fiber–matrix interfaces are represented by cohesive elements, so that high equivalent-stress bands in matrix ligaments and the associated crack paths can be resolved explicitly. Parametric analyses show that increasing fiber volume fraction raises the transverse elastic modulus and peak stress by thinning matrix ligaments and promoting longer, more continuous high-stress bands, while the cross-sectional aspect ratio redistributes high stress among ligaments and adjusts the balance between peak strength and the degree of failure localization. The observed size variability is represented by modeling the semi-minor axis as a normal random variable; a larger variance mainly leads to a reduction in transverse peak stress through stronger stress localization near very thin ligaments, whereas the elastic slope and the strain at peak stress remain almost unchanged. The proposed framework thus provides a statistically validated and computationally efficient micromechanical basis for microstructure-sensitive assessment of the transverse behavior of UD-CFRP plies with non-circular fiber cross-sections.

## 1. Introduction

Unidirectional carbon fiber-reinforced polymer (UD-CFRP) composites are widely used as lightweight load-bearing structural components for aerospace applications owing to their high specific strength and design flexibility [1,2,3]. In practical laminated structures, unidirectional plies are frequently subjected to transverse tensile loading in regions featuring notches, holes, joints, or local constraints. The transverse tensile strength obtained under this loading represents a fundamental mechanical property for assessing their transverse performance [4]. Under this loading condition, the resulting transverse stress–strain response is significantly influenced by the internal microstructure. The fiber packing morphology within the transverse cross-section and variations in fiber cross-sectional geometry can markedly modify local stress concentrations and crack initiation sites [5,6,7]. However, the influence of such microscopic geometric features on the transverse tensile failure process has not yet been systematically quantified.

In studies of transverse properties, micromechanical models with circular fiber cross-sections have long served as a basic modeling tool. Review work [8] has shown that representative volume element (RVE) models with circular fiber cross-sections are widely employed to generate fiber distributions and to predict transverse properties. These models have yielded systematic results across a range of numerical methods and loading cases. Within this framework, Naya et al. [9] employed micromechanical RVE models containing fibers with circular cross-sections, incorporating polymer matrix nonlinearity and interface damage to compute the transverse stiffness and strength of unidirectional plies. Comparison with experimental results confirmed the effectiveness of this micromechanical modeling framework. Under the assumption of circular fiber cross-sections, Alhaddad et al. [10] constructed a virtual material testing framework to assess the predictability of transverse tensile strength. Vaughan et al. [11] incorporated nonlinear matrix response into an RVE model with circular fiber cross-sections, whereas Yang et al. [12] employed RVE models with randomly distributed circular fibers and explicitly modeled fiber-matrix interface debonding under periodic boundary conditions. Overall, existing work indicates that micromechanical RVEs with an idealized uniform circular fiber cross-section can provide reasonably accurate predictions of the transverse tensile response of unidirectional plies, provided that the matrix and interface behavior are modeled appropriately.

However, microscopic observations and statistical analyses have shown that carbon fiber cross-sections in UD-CFRP composites are often elliptical or near-elliptical. The semi-minor axis length and cross-sectional area follow statistical distributions across fibers, and both ellipticity and characteristic dimensions exhibit pronounced scatter [13,14,15]. Considering these morphological features, previous work [16] introduced elliptical fiber cross-sections with prescribed aspect ratios into micromechanical models to examine the flattening effects on local stress and strength. Other studies have superimposed fiber-radius fluctuations on an underlying circular cross-section idealization to assess how size scatter influences stress concentrations and failure mechanisms. Relevant numerical and experimental results indicate that deviations from a circular fiber cross-sectional shape and fluctuations in fiber radius both modify the distribution of highly stressed regions within the transverse cross-section and the paths of crack propagation [7,17,18], thereby affecting the transverse tensile strength. However, these studies are generally conducted at a fixed fiber volume fraction and primarily focus on a single geometric factor. As a result, existing frameworks still struggle to represent fiber volume fraction, cross-sectional ellipticity, and size scatter at the same time, and to compare their relative effects on transverse tensile failure.

To address these limitations, a parametric micromechanical framework with elliptical fiber cross-sections is constructed for analyzing the transverse tensile response of UD-CFRP plies. The primary objective is to provide a consistent relative comparison of how fiber volume fraction, cross-sectional aspect ratio, and semi-minor-axis variability influence stiffness, peak stress, and failure localization, rather than to deliver a calibrated absolute strength prediction for a specific material system. Accordingly, the results are presented mainly in terms of trends and parametric sensitivities. The proposed framework differs from conventional circular-section models in three main aspects: (1) a unified geometric parameterization is introduced in which the fiber volume fraction, the cross-sectional aspect ratio and the statistical scatter of the semi-minor axis are treated as independent variables under the same material model and loading/boundary conditions, enabling a systematic examination of how different geometric combinations influence transverse stiffness, peak stress and the degree of failure localization; (2) a statistically validated RVE-generation procedure is employed, based on random sequential adsorption with a minimum edge-to-edge gap constraint and quantified by nearest-neighbor statistics, Ripley’s K function and the radial (pair) distribution function, so that the in-plane elliptical fiber arrangements remain compatible with experimentally observed random microstructures at relatively high fiber volume fractions; and (3) a finite element model with a damage-plasticity matrix and cohesive fiber-matrix interfaces, combined with local mesh refinement near elliptical fiber ends and the thinnest matrix ligaments, is used to perform virtual transverse tensile tests over a wide range of geometric parameter combinations, allowing the morphology and connectivity of high equivalent-stress bands in matrix ligaments to be directly related to the macroscopic stress–strain curves and to the observed patterns of crack initiation, propagation and coalescence. Together, these components form a unified and statistically validated parametric workflow. This integrated setting facilitates like-for-like trend-level comparisons of the relative effects of fiber volume fraction, aspect ratio and semi-minor-axis variability within ranges informed by microscopy observations and reported microstructural statistics, which is difficult to achieve when these geometric features are investigated separately.

The main framework of this manuscript is as follows: Section 2 presents the generation of an RVE with elliptical fiber cross-sections and its statistical validation. Section 3 describes the micromechanical finite element modeling and loading setup. Section 4 examines the effects of the three geometric parameters within a unified analysis framework, and Section 5 summarizes the main conclusions.

## 2. Micromechanical Modeling and Parameterized RVE Generation

A parametric procedure is established for generating RVEs with elliptical fiber cross-sections. Taking the semi-minor axis as the primary geometric variable, the fiber volume fraction and scatter in the semi-minor axis are controlled by adjusting its mean and variance, while the cross-sectional aspect ratio controls the semi-major axis length and the degree of ellipticity. Elliptical fibers are then placed within the RVE cross-section using a random sequential adsorption (RSA) algorithm with a minimum edge-to-edge gap constraint, ensuring that the surface-to-surface distance between any two fibers does not fall below a prescribed threshold. When the iterative insertion process has approached the target fiber volume fraction and the acceptance rate of new trial fibers has dropped markedly, the fiber center positions are kept fixed and the semi-minor axes of all fibers are uniformly scaled in a single step, so that the resulting fiber volume fraction is brought into the vicinity of the target value.

### 2.1. Generation Rules for RVEs with Elliptical Fiber Cross-Sections

The generated rectangular RVE has an in-plane side length of $L_{x}$ × $L_{y}$, with the fiber axial direction aligned with the thickness direction and only the fiber and matrix phases distinguished in the cross-section. The cross-section of an individual fiber is approximated as an ellipse, with the major and minor semi-axes denoted by *a* and *b*, respectively. Microscopic statistics indicate that, when the cross-section deviates from a circle, the deviation is mainly manifested as flattening or asymmetry along the major axis, whereas the fluctuation in the minimum diameter is comparatively small [19]. Accordingly, the semi-minor axis is taken as the reference dimension in the elliptical parameterization, and the fiber orientation is defined as the angle between the major axis of the ellipse and the x-direction. In the transverse cross-section, the overall microstructure is determined by the RVE dimensions, the fiber volume fraction, and a set of fiber cross-sectional shape parameters. The aspect ratio is defined as follows:

(1) $$q=\;\frac{b}{a}$$

To quantify how far the elliptical cross-section deviates from a circle. The semi-minor axis is assumed to follow a normal distribution with a mean of $b_{0}$ and a variance in $\sigma_{b}^{2}$. Denoting the maximum admissible value of *b* by $b_{max}$, one obtains:

(2) $$b_{max}\;=\;b_{0}\;+\;3\sigma_{b}$$

where a safety factor of 3 is adopted, corresponding to approximately 99.7% of cross-sectional sizes falling within this range. Accordingly, the upper bound of the semi-major axis is taken as $a_{max}$ = $b_{max}$/q.

To obtain a fiber orientation distribution approximating statistical isotropy in the cross-section, uniform sampling of the fiber orientation angle over the interval [0, π) is adopted. By varying the combinations of $V_{f}$, *q*, $b_{0}$, and $\sigma_{b}^{2}$, the effects of different fiber volume fractions, degrees of ellipticity, and levels of semi-minor axis scatter on the stress–strain response and crack evolution can be systematically examined.

To avoid fiber overlapping and retain sufficient matrix ligaments between adjacent fibers, a minimum surface-to-surface distance constraint is imposed. Specifically, the minimum distance between the boundaries of any two elliptical fibers must be at least the prescribed threshold $d_{\min}$. The threshold is scaled with the fiber size and is defined as no smaller than a prescribed fraction of the mean semi-minor axis (e.g., $d_{\min}$ > 0.01 $b_{0}$). This parameterization prevents direct contact between adjacent elliptical fiber cross-sections and mitigates the adverse effects of excessively narrow matrix ligaments on mesh quality and the post-peak softening response.

Under the above parameter settings and geometric constraints, fiber placement in the RVE follows an RSA algorithm. For a given parameter set ($L_{x}$ × $L_{y}$, $V_{f}$, *q*, $b_{0}$, $\sigma_{b}^{2}$, $d_{\min}$), fiber placement starts from an empty RVE and proceeds by repeatedly performing the following steps. First, a value of the semi-minor axis $b_{i}$ is sampled based on the reference value $b_{0}$ and $\sigma_{b}^{2}$, and the corresponding semi-major axis $a_{i}$ is determined from the prescribed aspect ratio *q*. The fiber center coordinates ($x_{i}$, $y_{i}$) are then sampled independently, with $x_{i}$ drawn uniformly from [0, $L_{x}$) and $y_{i}$ drawn uniformly from [0, $L_{y}$). Finally, the orientation angle $\theta_{i}$ is sampled from the interval [0, π), forming a candidate trial ellipse. The candidate fiber is checked against all previously accepted elliptical fibers by evaluating the minimum edge-to-edge distance between the candidate and each accepted fiber. If the minimum edge-to-edge distance to every accepted fiber is no smaller than $d_{min}$, the candidate fiber is accepted and added to the set. Otherwise, the candidate placement is rejected as violating the geometric constraint, and a new center position and orientation are resampled for the same fiber.

### 2.2. Generation of RVEs with Elliptical Fiber Cross-Sections

**Figure 1:**
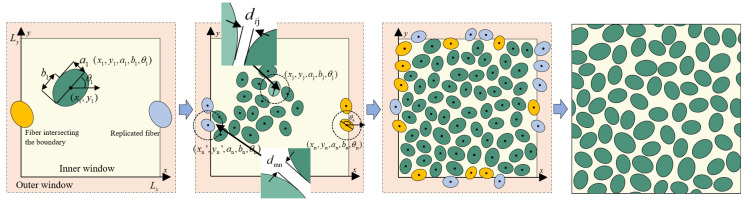
Flowchart for generating master RVE.

Given an RVE size $L_{x}$ × $L_{y}$, a target fiber volume fraction $V_{f}$, a prescribed minimum edge-to-edge distance $d_{\min}$, a semi-minor axis that follows a normal distribution with mean $b_{0}$ and variance $\sigma_{b}^{2}$, and a prescribed fiber aspect ratio *q*, the RVE generation procedure can be divided into the following steps. The overall workflow is summarized in Figure 1.

Step 1: An inner rectangular domain of size $L_{x}$ × $L_{y}$ is defined on the RVE cross-section, and an outer rectangular domain of size ($L_{x}$ + 2$\sqrt{2}a_{max}$) × ($L_{y}$ + 2$\sqrt{2}a_{max}$) is constructed with the same center. Whenever an accepted fiber intersects the inner domain boundary, a corresponding periodic fiber is generated at the opposite boundary.

Step 2: The initial fiber volume fraction is $V_{f}^{\left(0\right)}$ = 0. For the first fiber, the center coordinates ($x_{1}$, $y_{1}$) are uniformly sampled within the inner window. The semi-minor axis $b_{1}$ is sampled from a normal distribution with mean $b_{0}$ and standard deviation $\sigma_{b}^{2}$, and the semi-major axis is then given by $a_{1}=\;b_{1}/q$. The fiber orientation $\theta_{1}$ is uniformly sampled from the interval [0, π). The current fiber volume fraction is then updated as follows:

(3) $$V_{f}^{\left(1\right)}=\;\frac{\pi b_{1}^{2}}{qL_{x}L_{y}}$$

Step 3: Using the same sampling rules as for the first fiber, the geometric parameters of the second candidate fiber ($x_{2}$, $y_{2}$, $a_{2}$, $b_{2}$, $\theta_{2}$) are generated. First, the distance $r_{12}$ between the two fiber centers ($x_{1}$, $y_{1}$) and ($x_{2}$, $y_{2}$) is computed. When $r_{12}$ satisfies $r_{12}$ > ($a_{max}$ + $a_{2}$) + $d_{min}$, the actual edge-to-edge gap between the two ellipses is guaranteed to be no smaller than $d_{min}$, and the geometric constraint is satisfied; otherwise, the minimum edge-to-edge distance between the two elliptical boundaries is evaluated explicitly. The boundary of the *i*-th fiber can be expressed as follows:

(4) xit, yit= xi, yi+Rθibiqcostbisint t ϵ [0, 2π)

where ($x_{i}$(*t*), $y_{i}$(*t*)) denotes the boundary coordinates of the *i*-th elliptical fiber, *t* is the angular parameter sweeping from 0 to 2π to trace the entire ellipse, ($x_{i}$, $y_{i}$) are the coordinates of the fiber center, and *R*($\theta_{i}$) is the two-dimensional rotation matrix that rotates the standard ellipse by the orientation angle $\theta_{i}$ to obtain the boundary at the prescribed fiber orientation. The nearest distance between the first and second fibers is given by:

(5) $$d_{12}=min\left\|\left(x_{1}\left(t\right),\;y_{1}\left(t\right)\right)\;-\;\left(x_{2}\left(t\right),\;y_{2}\left(t\right)\right)\right\|$$

If $d_{12}$ > $d_{min}$, the second fiber is accepted and the current fiber volume fraction is updated. Otherwise, the coordinates of the second fiber are resampled. When the second fiber is accepted, the current fiber volume fraction is updated as follows:

(6) $$V_{f}^{\left(2\right)}=\;\frac{\sum_{i\;-1}^{2}\pi b_{i}^{2}}{qL_{x}L_{y}}$$

Step 4: For the *j*-th fiber lying entirely within the inner window, the placement proceeds as follows: First, the geometric parameters ($x_{j}$, $y_{j}$, $a_{j}$, $b_{j}$, $\theta_{j}$) are sampled according to the previously described rules. Next, all accepted fibers whose centers fall within a circle of radius ($a_{max}$ + $a_{j}$) + $d_{min}$ centered at ($x_{j}$, $y_{j}$) are identified, and their distances to ($x_{j}$, $y_{j}$) are evaluated using Equation (5). These distances are then compared with the prescribed minimum edge-to-edge distance $d_{min}$. If $d_{ij}$ > $d_{min}$ holds for all such neighboring fibers, the *j*-th fiber is accepted and the current fiber volume fraction is updated as $V_{f}^{\left(j\right)}$; otherwise, the candidate fiber is rejected and its position and geometric parameters are resampled.

For the *n*-th candidate fiber that may intersect the inner window boundary, the placement proceeds as follows: First, the geometric parameters ($x_{n}$, $y_{n}$, $a_{n}$, $b_{n}$, $\theta_{n}$) are sampled according to the previously described rules. Using Equation (4), boundary points on the corresponding ellipse are computed. If any of these points intersects or extends beyond the inner window boundary, the fiber is classified as a boundary fiber. In this case, a corresponding image fiber $n_{1}$ with geometric parameters ($x_{n_{1}}$, $y_{n_{1}}$, $a_{n_{1}}$, $b_{n_{1}}$, $\theta_{n_{1}}$) is generated on the opposite side by applying a translation vector (±$L_{x}$, 0) or (0, ±$L_{y}$). The minimum edge-to-edge distance between each of fibers *n* and $n_{1}$, all previously accepted fibers is then evaluated. The *n*-th fiber and its image are accepted only if, for both fibers, the minimum edge-to-edge distance to every accepted fiber is no smaller than $d_{min}$. Otherwise, the candidate fiber is discarded and its center coordinates are resampled. In updating the fiber volume fraction, only the cross-sectional areas of fibers whose centers lie inside the inner window are taken into account.

**Figure 2:**
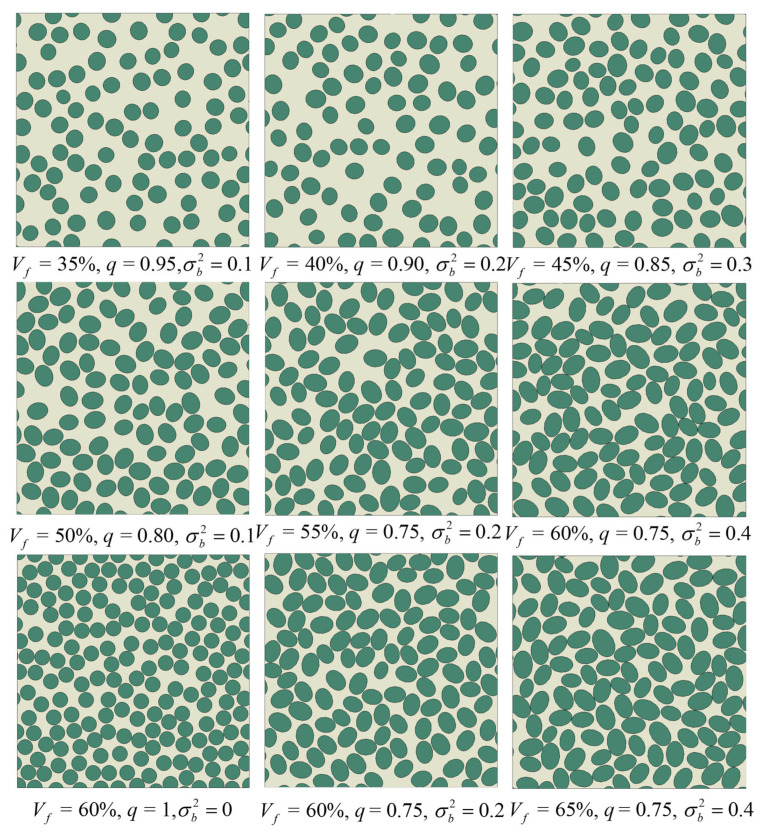
Generating RVE configurations with different $V_{f}$, *q* and $\sigma_{b}^{2}$.

Step 5: Subsequently, the above placement procedure is repeated fiber by fiber until the fiber volume fraction reaches $\left|V_{f}^{\left(n\right)}\;-\;V_{f}\right|$ ≤ 0.3%, at which point the microstructure generation is considered complete. If the fiber volume fraction is within the range 0.3% < $\left|V_{f}^{\left(n\right)}\;-\;V_{f}\right|$ ≤ 1% and repeated attempts to insert additional fibers are rejected by the geometric constraints so that the number of rejected trials reaches 200, a single small global scaling step is applied. In this step, all fibers are scaled by a factor $\sqrt{V_{f}/V_{f}^{\left(n\right)}}$ in the semi-minor axis while keeping their center coordinates fixed, so that the semi-minor axes are uniformly enlarged and the remaining gap to the target fiber volume fraction is closed without altering the spatial distribution of fiber centers. This geometry file is then parsed by an in-house Python (Version 3.7) script and used to build the finite element model in Abaqus/CAE. Representative RVE realizations generated by this procedure are shown in Figure 2.

## 3. Micromechanical Finite Element Modeling and Validation

### 3.1. Constitutive Damage Model and Material Parameters

The micromechanical finite element model consists of three parts: fibers, matrix, and the fiber-matrix interface. Under transverse tensile loading, the fiber strength is much higher than that of the matrix and the interface; so, the fibers primarily constrain the matrix deformation and transfer load. Accordingly, they are modeled as isotropic linear-elastic materials [16,20]. The matrix phase is defined as a transversely isotropic elasto-plastic material [21]. Epoxy resins typically exhibit pronounced nonlinear responses and damage accumulation under transverse tension [9,22,23]. The curvature concentration at the ends of elliptical fibers induces high hydrostatic pressure in the matrix and has a significant influence on damage initiation [7]. To capture these effects, a coupled damaged plasticity constitutive model is adopted for the matrix [24], which accounts for pressure sensitivity, tension–compression asymmetry, and stiffness degradation associated with damage evolution. The mechanical properties of the matrix and fibers are taken from Refs. [9,21,25,26,27], and the key parameters are summarized in Table 1.

The fiber-matrix interface is the primary site for the initiation of transverse cracking and debonding [28]. The interface is modeled using zero-thickness cohesive elements governed by a traction–separation law [29,30,31], and damage initiation is governed by a quadratic nominal stress criterion [32]. Damage evolution follows the Benzeggagh–Kenane (BK) mixed-mode fracture energy model [33]. The interfacial strengths and fracture energy parameters are taken from Refs. [20,25] with specific values provided in Table 1. To alleviate convergence difficulties associated with softening behavior and stiffness degradation, viscous regularization is implemented in the cohesive model [24], thereby ensuring that the tangent stiffness in the softening regime remains positive during each time increment.

Before applying mechanical loading, an initial analysis step is conducted that linearly reduces the uniform model temperature from the curing temperature of 180 °C to a room temperature of 20 °C, in order to introduce the thermal residual stresses induced by cooling from the curing temperature [34].

Periodic boundary conditions (PBC) are imposed on the RVE using the Abaqus plug-in EasyPBC to simulate the micromechanical response of the unidirectional composite under transverse tensile loading. These conditions constrain the relative displacements of nodes on opposite boundaries, ensuring periodic continuity of the microscopic stress and strain fields and thereby reducing boundary effects associated with the finite RVE size. The periodic boundary conditions can be written as follows:

(7) $$u_{x}^{R}\;-\;u_{x}^{L}=\;\varepsilon_{xx}L_{x}$$

(8) $$u_{y}^{R}-u_{y}^{L}=0$$

where *x* denotes the geometric length of the RVE in the x-direction and $\varepsilon_{xx}$ is the applied nominal transverse strain. In addition, a rigid-body translation constraint is applied to one boundary, and the rotational degree of freedom of a corner node is constrained to suppress rigid-body motion. The prescribed transverse displacement, corresponding to a nominal strain of 2%, is ramped linearly from 0 to its final value, and adaptive time stepping is enabled during the material softening regime to improve convergence behavior. The loading and solution procedures are carried out in Abaqus/Explicit.

### 3.2. Microscopic FE Modeling Procedure and Boundary Conditions

At the microstructure generation stage, the geometry is controlled by the parameters ($L_{x}$, $L_{y}$, $V_{f}$, *q*, $b_{0}$, $\sigma_{b}$, $d_{min}$). Previous experimental surveys have reported that UD-CFRP composites typically exhibit fiber volume fractions between 40% and 60%, and that the carbon fiber diameter predominantly lies in the range 6.5–7.5 μm [24,35]. For random heterogeneous microstructures, the RVE with transverse dimensions of approximately fifteen times the fiber diameter is sufficient for the two-point correlation function and the nearest-neighbor distance distribution to reach statistical stationarity [36]. With a nominal fiber diameter of about 7 μm, a cross-sectional size of 100 μm × 100 μm is therefore adopted, which accommodates roughly 80–120 fiber cross-sections and achieves a balance between statistical representativeness and computational cost.

Microscopy observations reported an eccentricity of about e ≈ 0.7, corresponding to q ≈ 0.72 [14]. Microscopy-based diameter statistics further suggest a lognormal distribution with a variance of about 0.2 [37]. Therefore, *q* = 0.75, 0.80, 0.85, 0.90, 0.95, and 1.0 and $V_{f}$ = 35, 40, 45, 50, and 55% [35] are adopted to construct a series of elliptical-fiber RVEs, thereby bracketing the microscopy-observed ellipticity level while retaining the circular limit, in order to compare the relative influence of aspect ratio and fiber volume fraction on the transverse tensile response and to examine their effects on the stress–strain behavior and damage evolution. In addition, for the parameter subset with $V_{f}$ = 45% and 50% and *q* = 0.75–1.0, RVEs are further generated by prescribing the semi-minor axis lengths to follow normal distributions with variances of 0.0, 0.1, 0.2, and 0.3 [37] so that the influence of semi-minor-axis scatter can be assessed in a controlled manner while keeping the nominal fiber volume fraction and aspect ratio unchanged.

A two-dimensional plane-strain cross-sectional RVE is employed because it captures the dominant in-plane failure mechanisms under transverse tension, namely transverse matrix cracking and fiber–matrix interfacial debonding. For a unidirectional ply subjected to macroscopic transverse tension, the fiber length is much larger than the transverse dimensions and the surrounding plies provide substantial through-thickness constraint, so that the stress state in the ply interior is close to plane strain. Under such constrained conditions, the stress concentrations that initiate failure are governed primarily by cross-sectional geometric features, including matrix-ligament thickness and the local curvature associated with fiber ellipticity, and similar two-dimensional cross-sectional RVEs have been widely used for analyzing the transverse response and microscopic damage of unidirectional plies [38,39,40]. Because plane strain implies a relatively high constraint level and stress triaxiality, the predicted initiation strength can be conservative in a quantitative sense. Nevertheless, the relative trends and parametric sensitivities, including the reductions caused by increasing ellipticity and by greater semi-minor-axis variability, are governed primarily by in-plane cross-sectional geometry and are therefore expected to remain robust within laminate interior regions under strong through-thickness constraint.

The cross-sectional modeling procedure is divided into two stages. In the first stage, the microstructure is generated in an external Python environment (Version 3.7) according to the prescribed geometric parameters. In the second stage, the geometric data file is imported into Abaqus/CAE to perform geometric reconstruction, definition of phase regions, mesh generation, and boundary-condition assignment. The external Python script calls the random sequential adsorption algorithm described in Section 2.2 to generate an arrangement of elliptical fibers that satisfies the minimum gap constraint and closely matches the target fiber volume fraction under the given cross-sectional size and target $V_{f}$. The center coordinates, semi-major and semi-minor axes, and orientation of each fiber are written to a data file in the form ($x_{i}$, $y_{i}$, $a_{i}$, $b_{i}$, $\theta_{i}$), which serves as the geometric input for the finite element model.

**Figure 3:**
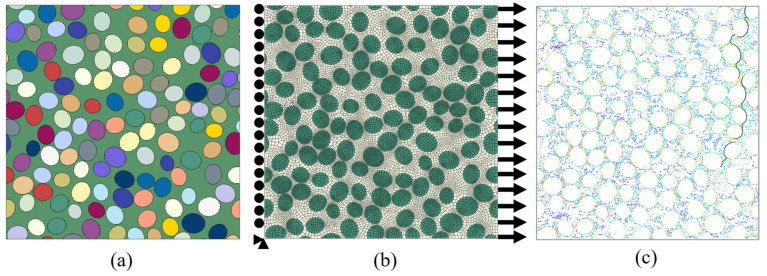
Overall procedure for microstructure generation, finite element modelling, and transverse tensile loading. (**a**) Generate microstructure; (**b**) Mesh generation and imposition of boundary conditions; (**c**) Crack propagation process.

The Abaqus/CAE Python script is employed to convert the geometric data file into a micromechanical finite element model. The script first draws the elliptical boundary of each fiber within the prescribed cross-sectional region to obtain the fiber phase and then uses Boolean operations to determine the remaining matrix phase region (Figure 3a). Mesh seeds are subsequently assigned to the fiber and matrix regions according to the mesh discretization strategy described in Section 3.3, and the mesh is generated. On this basis, the interfacial edges along the fiber-matrix boundary are identified and used to define an interface region, within which a layer of zero-thickness cohesive elements is created to discretize the fiber/matrix interface (Figure 3b). Material parameters and constitutive models listed in Table 1 are then assigned separately to the fiber, matrix, and interface regions. Finally, periodic displacement constraints are applied to opposite boundary nodes using EasyPBC, and cooling from the curing temperature followed by transverse displacement loading is imposed in the analysis steps, from which the transverse stress–strain curves and the evolution of microscopic damage and cracking are obtained (Figure 3c).

### 3.3. Mesh Discretization and Local Refinement Strategy

**Figure 4:**
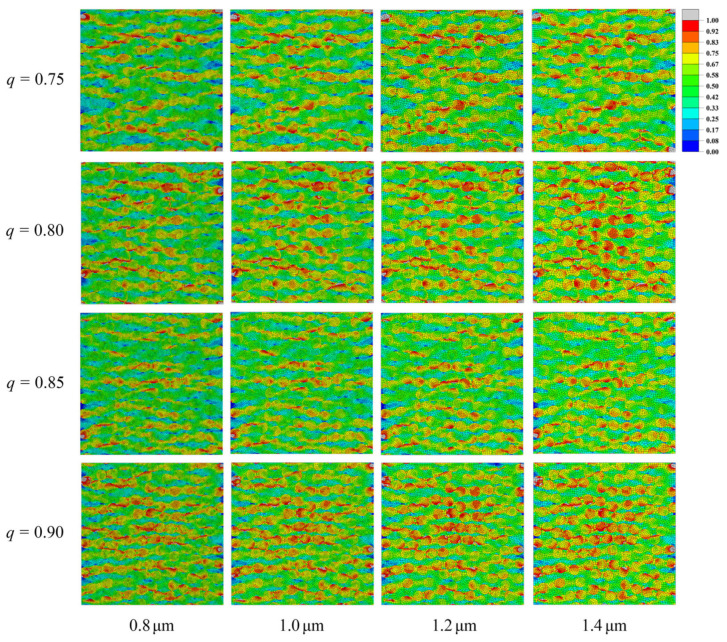
Effect of global mesh size on stress distribution for different aspect ratios.

To determine an appropriate global element size for the transverse tensile analysis, the RVEs with cross-sectional dimensions of 100 μm × 100 μm, $V_{f}$ = 50%, *b* = 3.5 μm, and *q* = 0.75, 0.80, 0.85, 0.90 is generated, as shown in Figure 4. Four global element sizes of 0.8, 1.0, 1.2 and 1.4 μm are examined, and the peak stresses of the resulting transverse stress–strain curves are compared. The results show that, for all values of *q*, the meshes with global element sizes of 1.0 μm and 0.8 μm produce almost identical peak stresses, with relative errors below 0.4%. Hence, refining the global element size from 1.0 μm to 0.8 μm has a negligible influence on the peak stress and stiffness. In contrast, the meshes with global element sizes of 1.2 μm and 1.4 μm show larger errors relative to the 0.8 μm reference mesh for all *q*, with the largest relative error in peak stress of approximately 0.9% observed at *q* = 0.80. Considering both accuracy and computational cost, the subsequent analyses use a global element size of 1.0 μm. On this basis, the meshing procedure is divided into a global mesh layout and two types of local refinement, as detailed below.

**Figure 5:**
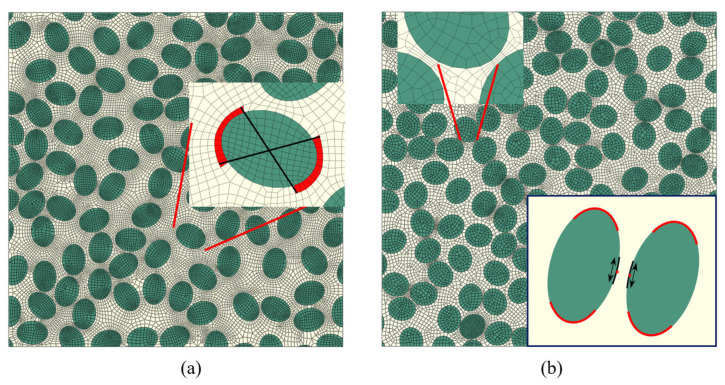
Local mesh refinement strategy in the elliptical-fiber RVE: (**a**) refinement near elliptical fiber ends; (**b**) refinement in matrix ligaments between fibers.

A global element size of 1.0 is adopted for the transverse tensile analyses, as indicated by the convergence study in Figure 5. The four outer edges of the RVE are partitioned into equal segments so that opposite edges share the same number of nodes, which simplifies the application of periodic displacement boundary conditions.

Local mesh refinement is restricted to regions that mainly control crack initiation, namely elliptical fiber ends and the thinnest matrix ligaments between neighboring fibers. Around each elliptical fiber end, a short angular sector of the outer contour centered at the major-axis tip is marked as a high-curvature end region, and mesh seeds with a target element size of 0.5 μm are assigned along the corresponding arc. Fiber ends that lie very close to the RVE boundary are not refined, so that unnecessary local over-refinement near the outer edges is avoided; outside the end regions, the global element size of 1.0 μm is retained.

For matrix ligaments, refinement is activated only for pairs of neighboring fibers whose minimum edge-to-edge distance falls below a prescribed threshold. For such pairs, the closest points on the two perimeters are identified, and short arc segments around these points are used to construct a small refinement patch enclosing the ligament. Mesh seeds with a target element size of 0.5 μm are placed along the boundary of this patch, focusing the refinement on the ligament segment expected to carry the highest equivalent stress. When a ligament refinement patch overlaps a fiber-end region, a ligament-priority rule is applied: the ligament refinement is kept and the overlapping part of the fiber-end refinement is suppressed, preventing excessive local mesh density and conflicts between mesh seeds. The final finite element discretization of the RVE cross-section is then generated from this combination of a uniform global mesh and geometry-driven local refinement.

### 3.4. Verification

#### 3.4.1. Model Validation

**Figure 6:**
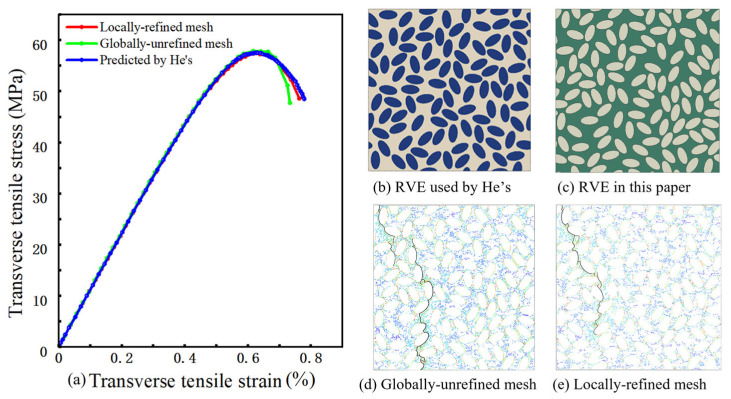
Model verification and comparison of mesh refinement strategies: (**a**) predicted transverse stress–strain curves compared with reference results; (**b**,**c**) corresponding RVE microstructures; (**d**,**e**) predicted crack paths under transverse tensile loading for different mesh refinement strategies.

To assess the reliability of the mesh discretization strategy, a set of microstructures is constructed with the same fiber volume fraction and RVE size as those used in He et al. [16] and the resulting transverse stress–strain curves are compared with the numerical results reported therein. The RVE cross-sectional dimensions are 64 μm × 64 μm, with $V_{f}$ = 50%, semi-minor axis *b* = 3.5 μm, aspect ratio *q* = 2, and minimum edge-to-edge distance $d_{min}$ = 0.2 *b*. The material parameters of the carbon fibers, epoxy matrix, and interface are identical to those reported in Ref. [16]. The corresponding transverse stress–strain curves are shown in Figure 6. The three curves exhibit virtually identical slopes in the initial linear regime, indicating that both discretization schemes predict the overall stiffness in close agreement with the reference solution. In the vicinity of the peak load, the strain values at peak stress and the peak stress levels are also almost the same for all three curves. The relative error in peak stress does not exceed approximately 0.6%, and the strain at peak stress lies in the range 0.62–0.63%, indicating that both meshing strategies accurately reproduce the strength level and peak characteristics of the reference curve.

To quantify the differences at the onset of post-peak softening, the transverse strain when the stress has dropped from its peak value to 50 MPa on the descending branch is compared. At this stress level, the transverse strains obtained from the reference curve, the locally refined mesh, and the mesh using only the global element size are approximately 0.77%, 0.75%, and 0.72%, respectively. Compared with the reference result, the locally refined mesh produces a slightly smaller strain at this stress level, with a relative error of about 2%, while the evolution of the softening branch remains in close agreement with the reference curve. In contrast, the mesh employing only the global element size gives a strain reduced by about 5% at the same stress level, indicating an earlier onset of the descending branch and a steeper post-peak drop, and thus a more pronounced underestimation of the post-peak deformation capacity and residual load-carrying capacity.

These comparisons indicate that, without degrading the prediction of the overall stiffness and peak strength, the local refinement strategy based on geometric features more consistently reproduces the response near the peak and the early softening behavior, whereas the mesh using a single global element size exhibits a certain degree of “premature drop” in the post-peak stage. Consequently, locally refined meshes are adopted in the subsequent analyses to ensure convergence of the response in the vicinity of the peak and, under a manageable computational cost, to improve the accuracy of the simulated stress–strain response during the post-peak damage-evolution stage.

#### 3.4.2. Scatter of Predicted Peak Stress Across Realizations

**Figure 7:**
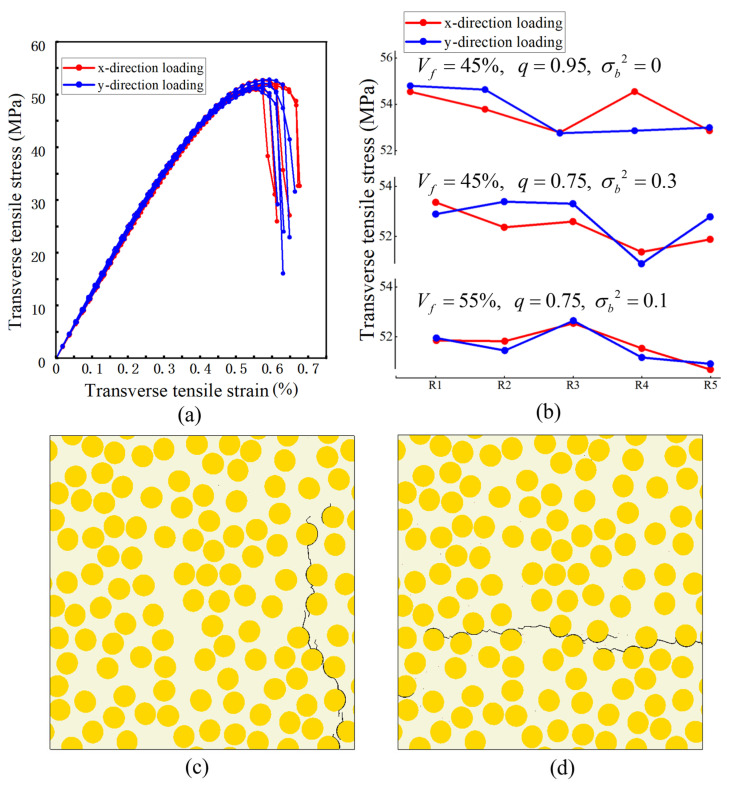
Realization-to-realization dispersion in predicted transverse tensile response and crack patterns. (**a**) Predicted stress–strain curves for $V_{f}\;$= 55%, *q* = 0.75, variance = 0.1 from multiple microstructure realizations. (**b**) Distribution of the predicted peak stress across microstructure realizations for selected parameter combinations. (**c**,**d**) Predicted crack patterns for $V_{f}\;$= 45%, realization R4, under two orthogonal in-plane loadings. Black lines denote the crack path.

At the ply scale, the transverse response of UD plies is commonly treated as approximately transversely isotropic in the plane normal to the fibers; therefore, x- and y-direction transverse loading are expected to yield broadly comparable responses in a statistical sense. To quantify the inherent variability introduced by random microstructural arrangement, an additional dispersion analysis was carried out on three representative parameter combinations: (i) fiber volume fraction 45%, aspect ratio 0.95, and zero semi-minor-axis variance; (ii) fiber volume fraction 50%, aspect ratio 0.75, and semi-minor-axis variance 0.3; and (iii) fiber volume fraction 55%, aspect ratio 0.75, and semi-minor-axis variance 0.1. For each combination, five independent RVE realizations (R1–R5) were generated using the same validated procedure, and transverse tension was applied along two orthogonal in-plane directions to avoid bias associated with an arbitrary coordinate choice. Figure 7a shows the stress–strain curves for the representative 55% case, where the pre-peak responses remain closely clustered across realizations. Figure 7b summarizes the peak stresses of all realizations for the three parameter sets. The coefficient of variation in peak stress across realizations remains low (about 1.3–1.9%), indicating limited realization-to-realization scatter for the examined cases. Representative crack patterns under the two orthogonal in-plane loadings are shown in Figure 7c,d, and the failure morphology remains dominated by interfacial debonding followed by matrix cracking through the thinnest ligaments.

These results quantify the realization-to-realization variability associated with stochastic microstructures. For the examined cases, the scatter of peak stress across five realizations remains below about 2% in coefficient of variation, which is smaller than the parametric differences discussed in Section 4. Therefore, unless otherwise stated, the stress–strain curves in Section 4 are shown for one representative realization for each parameter set for clarity.

## 4. Transverse Mechanical Response of UD-CFRP

### 4.1. Validation of Statistical Descriptors of the Fiber Distribution

**Figure 8:**
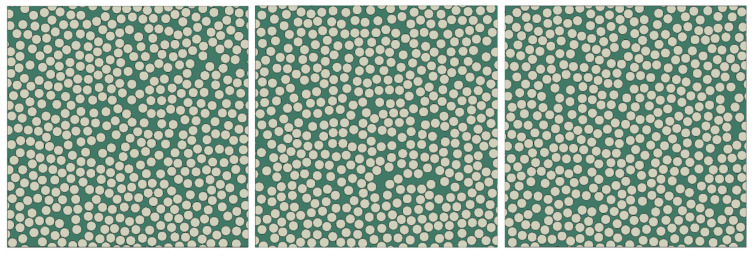
Examples of generated RVEs where ($L_{x}$, $L_{y}$, $V_{f}$, *q*, $b_{0}$, $d_{min}$) are set to (165 μm, 165 μm, 60% 1, 3.3 μm, 0, 0.1 μm).

To evaluate the capability of the microstructure generation algorithm to maintain spatial randomness at high fiber volume fractions, four commonly used statistical descriptors are employed: the nearest-neighbor distance distribution [41], the angular distribution of nearest neighbors [42], Ripley’s K function *K*(*r*) [42], and the radial distribution function *g*(*r*) [43]. To ensure statistical comparability with the results reported in reference [44], the input parameters for microstructure generation ($L_{x}$, $L_{y}$, $V_{f}$, *q*, $b_{0}$, $d_{min}$) are set to (165 μm, 165 μm, 60%, 1, 3.3 μm, 0.1 μm), where all length-type parameters are expressed in μm. A total of 20 independent RVE samples are generated, and three of these samples are shown in Figure 8.

#### 4.1.1. Nearest-Neighbor Distance

**Figure 9:**
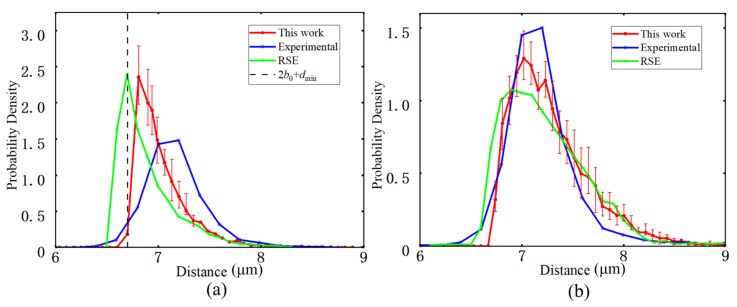
(**a**) The first nearest neighbor distance. (**b**) Second nearest neighbor distance.

The nearest-neighbor distance distribution characterizes the probability density of the distance from a given fiber to the center of its closest neighboring fiber [41], and is used to quantify short-range exclusion effects and local spatial uniformity. In the statistical analysis, each fiber center is taken as a reference point, the distances to all other fiber centers are computed, and the first and second nearest-neighbor distances are extracted to construct their empirical distributions, as shown in Figure 9.

Because a hard-core constraint with a minimum edge-to-edge distance of $d_{min}$ = 0.1 μm is imposed in the generation algorithm, the first nearest-neighbor distance distribution starts smoothly at approximately 2$b_{0}$ + $d_{min}$ = 6.7 μm, then develops a single dominant peak at larger distances and decays monotonically as the distance increases. No secondary peak or long tail is observed, indicating the absence of abnormal clustering or void regions at the local scale. Compared with the experimental results [37] and the theoretical curve obtained by the RSE method [44], the peak position for the generated model is close to that of the RSE curve. Both numerical curves exhibit slightly higher peak values and a peak position shifted to the right relative to the experimental curve. It implies that the typical fiber spacing in the numerical realizations is somewhat larger than in the actual material, consistent with previous analyses of ideal random hard-core models reported in the literature.

#### 4.1.2. Nearest-Neighbor Orientation

**Figure 10:**
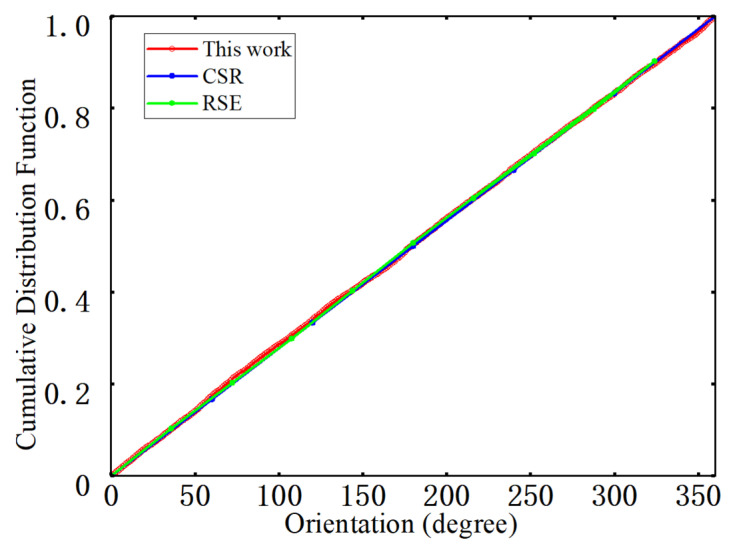
Nearest neighbor direction.

The angular distribution of nearest-neighbor fiber pairs in the cross-section is used to characterize the in-plane orientation of nearest-neighbor directions and to detect possible preferred directions or anisotropy [42]. In the statistical procedure, the angle between each nearest-neighbor vector and the horizontal axis is mapped into the interval [0, 360°), and the cumulative distribution function (CDF) is computed, as shown in Figure 10. The figure compares nearest-neighbor orientation CDFs from experiments, the RSE model, and the present generation algorithm. The three curves nearly overlap and are close to a straight line over the entire angular range, indicating that the nearest-neighbor orientations are approximately uniformly distributed in the plane without any pronounced preferred directions. The curve corresponding to the generation algorithm used here varies smoothly with only small fluctuations and remains consistent across the 20 RVE realizations, reflecting good directional randomness and statistical stability. Under the high fiber volume fraction considered, the generated microstructures therefore satisfy both the non-overlapping constraint with a prescribed minimum edge-to-edge distance and the requirement of random nearest-neighbor orientation, and the fiber arrangement can be regarded as statistically isotropic in the plane.

#### 4.1.3. Spatial Randomness Assessment Using Ripley’s K Function

**Figure 11:**
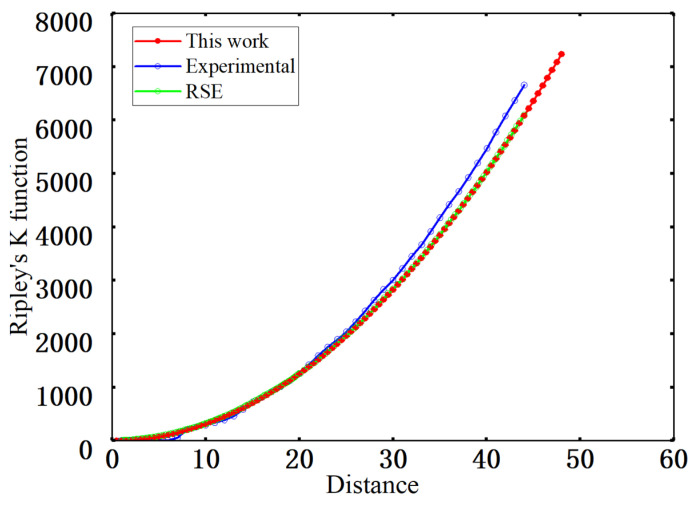
Ripley’s K function.

Ripley’s K function *K*(*r*) describes the expected number of fiber centers located within a distance *r* from an arbitrary fiber center in the point pattern and is used to characterize clustering or regularity at intermediate and larger length scales [42]. In this work, *K*(*r*) is evaluated from the fiber center coordinates and compared with the experimental data and the RSE-based reference curve, as shown in Figure 11. All three curves increase monotonically with and exhibit very similar trends. At short and intermediate ranges, the curve for the generated microstructures almost coincides with the experimental curve. Despite the imposed non-overlapping constraint with a minimum edge-to-edge distance, the local arrangements remain close to a random pattern. For distances larger than about 20 μm, the experimental curve lies slightly above the model and RSE curves, whereas the curve for the generated microstructures nearly overlaps the RSE curve, indicating that the numerically generated structures do not introduce additional clustering at intermediate and larger scales and that their statistics are closer to a completely spatially random (CSR) pattern. Overall, the *K*(*r*) curves agree well with the reference results overall length scales, with no evidence of excessive regularity or anomalous clustering, which confirms the statistical validity and spatial representativeness of the generated microstructures at high fiber volume fraction.

#### 4.1.4. Spatial Distribution Assessment Using the Radial Distribution Function

**Figure 12:**
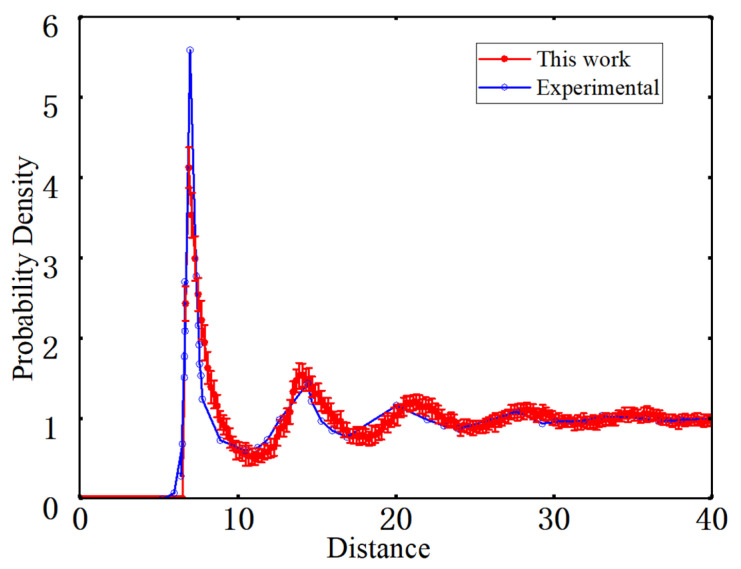
Radial distribution function.

The radial distribution function *g*(*r*) characterizes spatial correlations at different length scales from the viewpoint of a normalized number density [43]. It is obtained by normalizing the histogram of distances between all pairs of fiber centers, and the resulting *g*(*r*) curve is compared with the experimental result, as shown in Figure 12. The experimental and simulated *g*(*r*) curves exhibit a similar overall trend. Both display a pronounced peak at 7 μm, corresponding to the characteristic scale imposed by the minimum edge-to-edge spacing between fibers. The peak position of the simulated curve is slightly smaller than that of the experimental curve, mainly due to the use of ideal elliptical cross-sections and a uniform minimum-gap assumption in the geometric generation procedure.

As the distance increases, *g*(*r*) gradually decreases and approaches unity at 30 μm, with only minor fluctuations, indicating that the fiber distribution is close to random at intermediate and larger length scales. In addition, the *g*(*r*) curves obtained from the 20 RVE realizations lie within a narrow error band over the entire distance range, demonstrating a high level of consistency among different random realizations. Overall, apart from a slight shift in peak position, the radial distribution function of the generated microstructures agrees well with the experimental curve at intermediate and larger scales, indicating good consistency in both short-range exclusion and medium-range to long-range randomness and satisfying the spatial statistical requirements of subsequent micromechanical analyses.

### 4.2. Effect of Fiber Volume Fraction and Aspect Ratio

**Figure 13:**
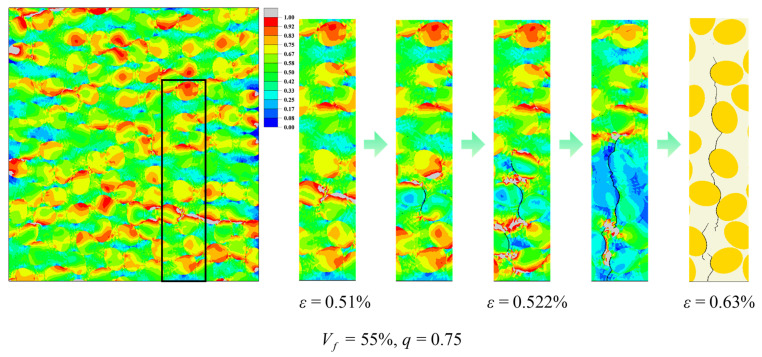
Predicted crack initiation and propagation in the elliptical-fiber UD-CFRP RVE under transverse tensile loading. The black rectangle marks the enlarged area.

As shown in Figure 13, under transverse tensile loading, high stress concentrations first appear in the matrix ligaments between adjacent fiber rows aligned perpendicular to the loading direction and in the neighboring interfacial layers. Local interface cracks subsequently nucleate along these regions and gradually coalesce within the cross-section, ultimately forming a main crack path that propagates across multiple inter-fiber ligaments. The combination of volume fraction and aspect ratio controls this initiation and growth process by modifying the number of high-stress regions, the lengths of matrix ligaments spanning the inter-fiber gaps, and their spatial distribution.

**Figure 14:**
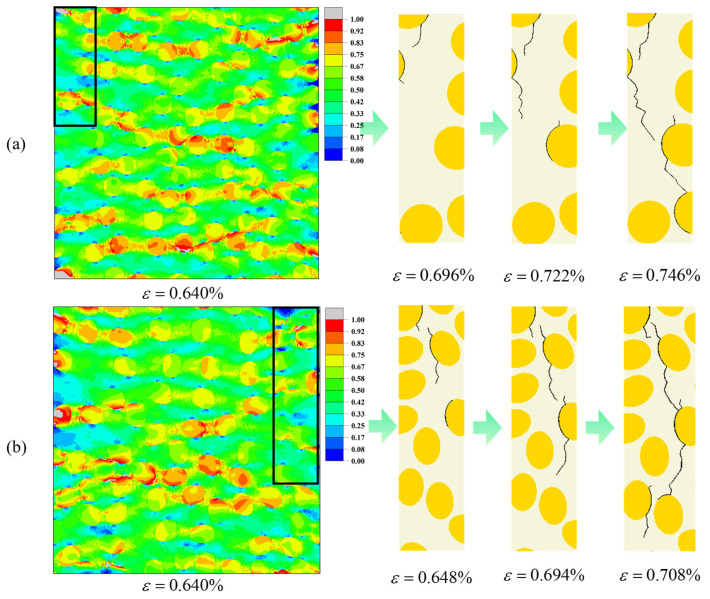
Predicted equivalent stress contours and crack paths of the elliptical-fiber UD-CFRP RVE under transverse tensile loading at $V_{f}$ = 35% (**a**) *q* = 0.95; (**b**) *q* = 0.75. The black rectangle marks the enlarged area.

For $V_{f}$ = 35% (Figure 14), changing the aspect ratio mainly redistributes high-stress regions without significantly altering the overall crack trajectory. When *q* = 0.95 (Figure 14a), high equivalent stress forms relatively wide and continuous band-like regions between fiber rows perpendicular to the loading direction, spanning multiple inter-fiber gaps and being distributed rather uniformly. When *q* is reduced to 0.75 (Figure 14b), the high equivalent stress is instead concentrated in a few thinnest matrix ligaments and in local neighborhoods near some elliptical fiber ends, and the high-stress regions appear as discrete patches or short bands. In both cases, crack evolution follows a similar pattern: fine cracks first nucleate in the highly stressed interfacial layers, then successively penetrate neighboring matrix ligaments, and finally coalesce into a main crack path that traverses the cross-section perpendicular to the loading direction.

**Figure 15:**
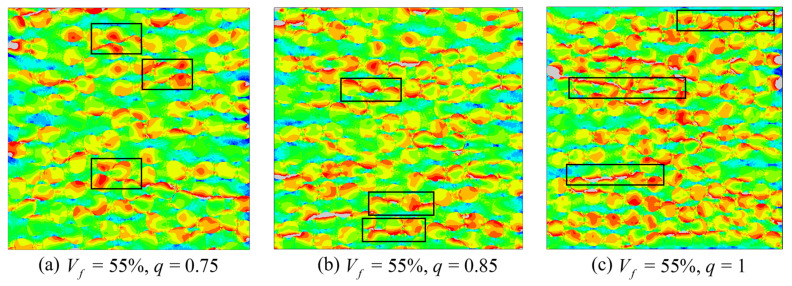
Predicted equivalent stress contours of the elliptical-fiber UD-CFRP RVE at $V_{f}$ = 55%. The black rectangles highlight representative high-stress band regions.

At $V_{f}$ = 55% (Figure 15), the matrix ligaments between fibers become thinner and both the continuity and density of high equivalent-stress regions increase. For *q* = 0.75 (Figure 15a), one prominent long high-stress band develops within the ligaments between fiber rows perpendicular to the loading direction, surrounded by numerous smaller compact high-stress patches. *Q* = 0.85 (Figure 15b), high equivalent stress forms band-shaped regions along portions of the fiber rows while also accumulating in block-like regions near elliptical fiber ends and local ligaments. For *q* = 1 (Figure 15c), the high-stress pattern is dominated by a few slender continuous bands that extend across multiple inter-fiber gaps, and scattered local high-stress patches are markedly reduced.

**Figure 16:**
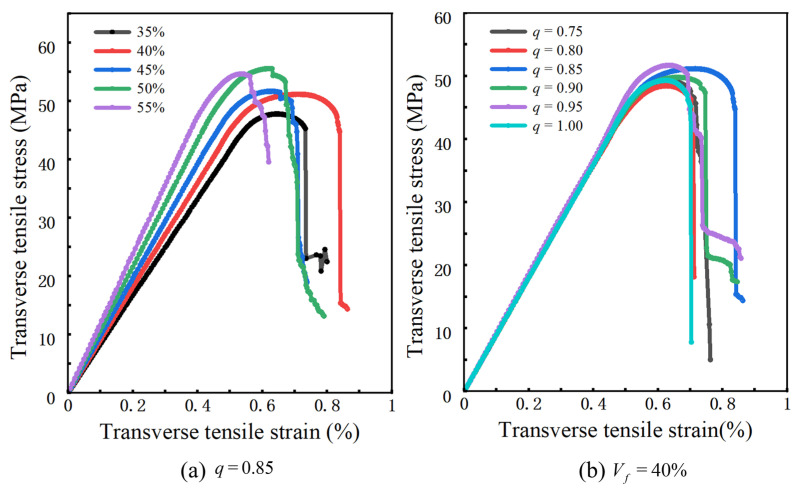
Predicted transverse stress–strain curves of the elliptical-fiber UD-CFRP RVE: (**a**) effect of fiber volume fraction; (**b**) effect of aspect ratio.

Figure 16a shows that for *q* = 0.85, as $V_{f}$ increases from 35% to 55%, all predicted stress–strain curves remain approximately linear in the small-strain regime. The slope of this linear segment and the peak stress increase simultaneously with $V_{f}$, whereas the strain at the peak exhibits only small variations. Consistent with the stress contour plots, increasing $V_{f}$ shortens and reduces the number of matrix ligaments between fibers, so that band-like high-stress regions become denser in the cross-section and develop longer connected paths. As a result, a larger portion of the load is carried by the fibers and the interfaces, leading to higher overall stiffness and peak strength at comparable deformation levels.

Figure 16b shows that, at $V_{f}$ = 40% and for (*q*) between 0.75 and 1.0, the transverse stress–strain curves are nearly indistinguishable in the elastic regime. Both the peak stress and the corresponding strain remain very similar across different aspect ratios, indicating that variations in cross-sectional shape have only a minor influence on the initial stiffness and peak position at this fiber volume fraction. The main effect of *q* appears in the onset of post-peak softening, as reflected by the equivalent-stress contour plots. For circular cross-sections and more strongly flattened ellipses, the high equivalent stress is concentrated within continuous band-like channels or a few extremely narrow matrix ligaments. Once a crack initiates, it tends to propagate rapidly along a single dominant path, and the stress drops sharply over a short post-peak strain interval. For intermediate aspect ratios (*q* ≈ 0.8–0.9), the high-stress regions are instead distributed over several matrix ligaments of moderate length. Crack growth proceeds through progressive linkage of multiple local damage zones, and the stress decay near the peak becomes noticeably more gradual.

### 4.3. Effect of Fluctuations in the Semi-Minor Axis

**Figure 17:**
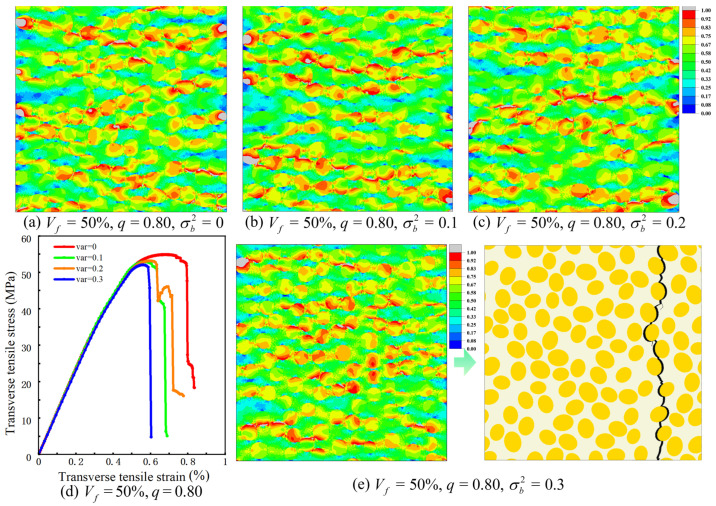
Predicted transverse response of the elliptical-fiber UD-CFRP RVE with semi-minor-axis variance: (**a**–**c**) equivalent stress contours for different variances in the semi-minor axis; (**d**) transverse stress–strain curves; (**e**) crack pattern at complete failure, the black regions indicate cracks.

Figure 17 shows the influence of semi-minor-axis variance on the transverse response for $V_{f}$ = 50% and *q* = 0.80. In this set of analyses, only the variance in the semi-minor axis is varied, with values of 0.0, 0.1, 0.2 and 0.3. The equivalent-stress contours in Figure 17a–c correspond to variances of 0.0, 0.1 and 0.2, respectively. In all three cases, high equivalent stress remains concentrated in the matrix ligaments between neighboring fibers and in the adjacent interfacial layers, and the main crack path still forms approximately normal to the loading direction. As the variance increases, the high-stress bands become slightly more localized around the thinnest ligaments and near a few fibers with more pronounced size deviation, but no additional high-stress channels or alternative crack trajectories are observed. The final crack pattern for the largest variance considered, illustrated in Figure 17e for a variance of 0.3, is similar to that of the configuration without semi-minor-axis fluctuations and is still governed by interfacial debonding followed by ligament failure.

The transverse stress–strain curves in Figure 17d reveal that the influence of semi-minor-axis variance is more clearly reflected in peak stress than in stiffness. All four curves, corresponding to variances of 0.0, 0.1, 0.2 and 0.3, share almost the same elastic slope, indicating that the transverse elastic modulus is essentially unaffected by the fluctuations. Introducing a nonzero variance leads to a marked reduction in peak stress compared with the case without fluctuations, whereas further increasing the variance from 0.1 to 0.3 produces an additional but more gradual decrease. The strain at peak stress changes only moderately over this range, and the overall shape of the post-peak softening remains similar. Taken together, these results indicate that, in the parameter range examined here, statistical fluctuations in the semi-minor axis primarily reduce the transverse peak strength through enhanced stress localization near a subset of very thin matrix ligaments, while the global stiffness and the overall failure mode remain close to those of the corresponding configuration with a deterministic semi-minor axis.

## 5. Conclusions

This work is designed as a relative parametric study rather than a calibrated predictive model for a specific material system. A two-dimensional plane-strain micromechanical RVE framework with elliptical fiber cross-sections was established for the transverse tensile analysis of unidirectional CFRP plies. Elliptical fiber arrangements were generated by a hard-core random sequential adsorption algorithm with a minimum edge-to-edge gap constraint, and the resulting microstructures were checked using nearest-neighbor distance statistics, Ripley’s K function and the radial distribution function. On this geometric basis, a three-phase finite element model with a damage–plasticity matrix, a cohesive interfacial layer and local mesh refinement near elliptical fiber ends and the thinnest matrix ligaments was used to perform virtual transverse tensile tests. By independently varying fiber volume fraction, aspect ratio and semi-minor-axis scatter within physically relevant ranges informed by microscopy and reported microstructural statistics, the framework decouples geometric effects that are inherently coupled in real materials and enables a consistent, like-for-like comparison of their relative effects on stiffness, peak stress and failure localization within the examined ranges.

Within the examined range of fiber volume fractions and aspect ratios, the simulations indicated that transverse stiffness and strength were closely linked to the thickness of matrix ligaments and to the formation of high equivalent-stress bands between fibers. Increasing fiber volume fraction shortened and thinned the ligaments, promoted longer and denser high-stress bands across multiple inter-fiber gaps, and led to higher transverse elastic modulus and peak stress, while the strain at peak stress changed only weakly. The predicted increase in transverse stiffness and peak stress with increasing fiber volume fraction is qualitatively consistent with commonly reported experimental trends for transversely loaded UD plies. In the present parametric results, this increase occurs together with thinner matrix ligaments and more connected high-stress bands. At a fixed fiber volume fraction, varying the aspect ratio between 0.75 and 1.0 mainly redistributed high-stress regions among ligaments and adjusted the degree of failure localization: nearly circular cross-sections and more strongly flattened ellipses tended to form one or a few dominant band-like channels and showed a relatively steep post-peak stress drop, whereas aspect ratios around 0.8 and 0.9 spread high-stress regions over several ligaments and produced a more gradual softening at a comparable peak stress within the parameter combinations considered.

Statistical variability in the semi-minor axis was introduced to represent observed fluctuations in fiber size. In the cases analyzed, increasing the variance in the semi-minor axis enhanced stress localization in a small subset of very thin matrix ligaments and caused a reduction in transverse peak stress compared with configurations without such fluctuations, while the transverse elastic modulus and the overall crack pattern remained similar and no new failure modes were observed. Under the present plane-strain and two-dimensional idealizations, the framework therefore provides a computationally efficient way to relate cross-sectional geometry and semi-minor-axis variability to transverse tensile behavior, with the pre-peak stiffness, peak stress and main crack paths offering more reliable quantitative information than the long post-peak softening segment. For the selected cases examined with multiple RVE realizations and orthogonal in-plane loading, the variability in peak stress remains small and is notably smaller than the parametric shifts discussed in Section 4, supporting the robustness of the trend-level conclusions.

It is important to acknowledge that the present micromechanical framework is built upon a two-dimensional plane-strain idealization. This approach remains a valid and efficient tool for probing the dominant in-plane failure mechanisms, such as transverse matrix cracking and fiber–matrix debonding, that are governed by fiber cross-sectional geometry, including ellipticity. Its predictions are most reliable for simulating the onset of transverse failure within ply-interior regions of laminates where strong out-of-plane constraint is present. Although absolute strength predictions may be influenced by three-dimensional stress effects, the relative trends and parametric dependencies of the transverse mechanical response on fiber geometric imperfections, such as aspect ratio and semi-minor-axis variability, are robust, because they are primarily governed by the in-plane geometric features of the ply cross-section. In this sense, the predicted failure morphology (interface debonding followed by matrix cracking and transverse crack formation) is consistent with reported microscopy observations under transverse tensile loading. The identified sensitivity of peak stress to geometric scatter indicates that small variations in fiber curvature can shift where the thinnest matrix ligaments occur and how they are distributed, which can contribute to transverse peak-stress scatter even at the same nominal fiber volume fraction. Future work may combine detailed microstructural characterization with inverse identification of cohesive parameters to facilitate material-specific calibration, and extend the framework to three-dimensional RVEs for cases with spatially varying out-of-plane constraint.

## Figures and Tables

**Table 1:** Material parameters of each component in the microstructure.

Component	Property				
Matrix	$E_{m}(\mathrm{MPa})$ 3760	$v_{m}$ 0.39	$v_{p}$ 0.3	$\sigma_{m,t}^{0}(\mathrm{MPa})$ 29	$\sigma_{m,c}^{0}(\mathrm{MPa})$ 67
	$\alpha_{f1}\left(\mathrm{GPa}\right)$ −3.15	$\alpha_{f2}\left(\mathrm{GPa}\right)$ 10.0074	$X_{ft}$(MPa) 4000	$X_{fc}$(MPa) 3500	
Carbon Fiber	$E_{f1}(GPa)$ 231	$E_{f2}\left(\mathrm{GPa}\right)$ 35	$G_{f12}\left(\mathrm{GPa}\right)$ 24	$G_{f23}$(GPa) 14.3	$v_{f12}$(N/mm) 0.26
	$\psi^{m}({}^{\circ})$ 29	$X_{m,c}$(MPa) 0.39	$X_{m,t}$(Mpa) 0.3	$n_{t}$ 170	$G_{m}$(N/mm) 0.09
Fiber-Matrix Interface	$\sigma_{n}^{0}(\mathrm{MPa})$ 7	$\tau_{s}^{0}(\mathrm{MPa})$ 85	$K_{n}$(MPa/mm) $10^{8}$	$K_{s}$(Pa/mm) $10^{8}$	$G_{n}^{c}$(mJ/mm^2^) 0.002
	$G_{n}^{s}$(mJ/mm^2^) 0.006	η 2			

## Data Availability

The original contributions presented in this study are included in this article. Further inquiries can be directed to the corresponding author.

## Abbreviations

The following abbreviations are used in this manuscript:

CFRP	carbon-fiber-reinforced polymer
UD-CFRP	unidirectional carbon-fiber-reinforced polymer
FRP	fiber-reinforced polymer
RVE	representative volume element
RSA	random sequential adsorption
RSE	random sequential expansion
FEM	finite element model
PBC	periodic boundary condition
CDF	cumulative distribution function
